# A National Observational Study of the Prevalence and Use of Enteral Tube Feeding, Parenteral Nutrition and Intravenous Glucose in Cancer Patients Enrolled in Specialized Palliative Care 

**DOI:** 10.3390/nu5010267

**Published:** 2013-01-22

**Authors:** Ylva Orrevall, Carol Tishelman, Johan Permert, Staffan Lundström

**Affiliations:** 1 Division of Surgery, Department of Clinical Science, Intervention and Technology, Karolinska Institutet, Karolinska University Hospital-Huddinge, SE-141 86 Stockholm, Sweden; E-Mail: johan.permert@karolinska.se; 2 Stockholms Sjukhem Foundation, Box 12230, SE-102 26 Stockholm, Sweden; E-Mails: carol.tishelman@ki.se (C.T.); staffan.lundstrom@stockholmssjukhem.se (S.L.); 3 Department of Learning, Informatics, Management and Ethics, Karolinska Institutet, SE-171 77 Stockholm, Sweden; 4 School of Nursing, Midwifery and Social Work, University of Manchester, Manchester M13 9PL, UK; 5 Department of Oncology-Pathology, Karolinska Institutet, SE-171 77 Stockholm, Sweden

**Keywords:** enteral tube feeding, intravenous glucose, neoplasm, nutritional support, palliative care, parenteral nutrition

## Abstract

The use of artificial nutrition remains controversial for cancer patients in palliative care, and its prevalence is largely unknown. We therefore conducted a national study to investigate the prevalence, indications for, and perceived benefit of enteral/parenteral nutrition and intravenous glucose in this patient group. A cross-sectional study was performed within the palliative care research network in Sweden (PANIS), using a web-based survey with 24 questions on demographics, prescribed nutritional treatment, estimated survival and benefit from treatment. Data was received from 32 palliative care units throughout the country, representing 1083 patients with gastrointestinal and gynecological malignancies being the most common diagnoses. Thirteen percent of the patients received enteral/parenteral nutrition or intravenous glucose. Parenteral nutrition (PN) was significantly more common in home care units serving the urban Stockholm region (11%) than in other parts of the country (4%). Weight and appetite loss were the predominant indications for PN, with this treatment deemed beneficial for 75% of the palliative patients. Data show that there was great variation in PN use within the country. PN was predominately initiated when patients had weight and appetite loss but still had oral intake, indicating a use of PN that extends beyond the traditional use for patients with obstruction/semi obstruction.

## 1. Introduction

About two thirds of cancer patients enrolled in palliative home care services have been found to be at nutritional risk [1,2]. Despite the fact that nutritional problems and weight loss are common in this group of patients [3,4], the use of artificial nutrition (enteral tube feeding (ETF) and parenteral nutrition (PN)) remains controversial [5,6] and the prevalence of such treatments is largely unknown. Data on the benefits and risks of nutritional interventions and nutritional support for patients in palliative stages of cancer is lacking [7]. The metabolic causes of weight loss in cancer patients including loss of fat and muscle mass are not yet fully understood, with the definition of cachexia much discussed in recent years [3,4,8,9]. A recent international consensus definition states that cancer cachexia is a “multifactorial syndrome defined by ongoing loss of skeletal muscle mass (with or without loss of fat mass) that cannot be fully reversed by conventional nutritional support and leads to progressive functional impairment” [4]. The definition further states that cachexia can develop progressively through three stages; precachexia, cachexia and refractory cachexia. 

Use of the term palliative care in research and practice varies between studies [10], although it was defined in 2002 by the WHO [11] as an approach that improves the quality of life of patients and their families facing problems associated with life-threatening illness. According to this definition, palliative care can be relevant in both early and late phases of disease. In early palliative phases, palliative care can be given in conjunction with other therapies intended to prolong life, *i.e.*, chemotherapy or radiation therapy. In late palliative phases, the care goals are solely focused on symptom relief and patient wellbeing. Survival times for patients with incurable cancer have been dramatically extended in recent years and many patients receiving specialized palliative care also receive antitumoral treatment to both prolong life and provide symptom relief [12]. This makes the transition between palliative and curative treatment increasingly diffuse, and means that clarification of treatment goals is essential as these differ by individual, disease, treatment opportunities as well as along the disease trajectory. This situation is further complicated by a lack of clinically relevant evidence to guide clinicians in decision-making about when and how to design nutritional interventions, especially regarding the use of ETF and PN in both earlier and later palliative phases [7,13]. Effects of nutritional support may vary depending on the underlying causes of the patient’s nutritional problem, but such causes are not specified in most studies. This is partly because clinically relevant methods to distinguish impaired nutritional status caused by cachexia from that caused by simple starvation and secondary symptoms are not well developed [3,7]. A Cochrane review from 2009 [14] concluded that there are insufficient good quality studies to make any recommendations for practice with regards to the use of ETF or PN in palliative care patients. Raijmakers *et al.*’s more recent review [15] on cancer patients in the last week of life supports this conclusion. Therefore, decisions need to be made on an individual basis taking into account the perceived benefits and risks to the patient. Similarly, evidence for continuing or withdrawing artificial nutrition in the last week of life is lacking [15]. One consequence of this is that the appropriate use of nutritional support in palliative cancer care is often subject to debate. In the current guidelines from the European Society for Clinical Nutrition and Metabolism [5] it is stated that PN may be recommended in incurable cancer patients who cannot be fed orally or enterally if it is estimated that they will die sooner from starvation than from tumor progression. Also, their performance status and quality of life should be acceptable and the patient and family should be motivated towards this demanding treatment. 

Despite the lack of solid evidence supporting the use of nutritional support in palliative phases, increasing use of home parenteral nutrition (HPN) for patients with cancer has been reported in many parts of Europe, although with great variation between countries [16]. However, data about the prevalence of ETF and PN in palliative phases is scarce and difficult to compare between different countries due to differences in the way nutritional support and palliative care are organized. 

As data regarding treatment with artificial nutrition in Sweden is not systematically registered beyond individual medical records, relatively little is known about its use among cancer patients in Swedish palliative care settings. In an earlier study [1,17], we investigated the prevalence and use of artificial nutrition in specialized palliative home care (PHC) services in the Stockholm area, from the perspective of patients and their family members [1,17,18,19]. Telephone interviews were conducted with over 600 patients with cancer and/or family members, showing that 11% of these patients received PN and 3% ETF [17]. These results suggested that the main indication for PN was to supplement oral intake of patients with weight loss and anorexia. The present national study complements our previous work, by providing national data from urban, rural and remote regions of Sweden based on reports by professional palliative care staff. 

The aim of the present study was to further investigate the prevalence and use of ETF, PN and intravenous glucose among patients diagnosed with cancer who were enrolled in specialized palliative in-patient and home care services throughout Sweden. An additional aim was to investigate the indications for and perceived benefit of artificial nutrition and intravenous glucose in relation to predicted survival, focusing on professional perspectives of these issues.

## 2. Materials and Methods

This study was performed through the Palliative Care Research Network in Sweden (PANIS). PANIS was established in 2002 and at the time of this study was comprised of 42 specialized palliative care units with physician-directed multi-professional teams available around-the-clock. Some services included both palliative in-patient units (PIU) as well as specialized PHC services. Mean life expectancy of patients in PIUs participating in the network is approximately two to three weeks and in PHCs two to three months. Only 10%–15% of patients in in-patient units are discharged to their homes or to other caring facilities.

PANIS conducts cross-sectional survey studies about symptom prevalence and treatment traditions in palliative care, and the working method has shown to be efficient in collecting clinical data from a large number of patients [12,20]. In palliative care this is important, since recruitment and data collection among patients with limited life expectancy is notoriously difficult [21]. All data is reported by the responsible clinician at each unit and is based on individual patient records and/or personal knowledge of patients. Patients’ self-assessments are not collected directly by PANIS. All patients enrolled in the unit are registered, so that patients referred in early palliative phases and those referred for supportive care in potentially curative stages will also be included in the data set. As there are no specific agencies providing artificial nutrition or intravenous glucose to patients in Sweden, this often falls under the remit of PHC as part of supportive care.

An invitation to participate in this study was sent to all units along with a survey on the use of ETF, PN and intravenous glucose. The survey consisted of 24 questions on age, gender, diagnosis, and, with regard to patients receiving treatment, specific multiple choice questions about prescribed treatment, goals of care, estimated survival, and benefit from treatment. The study was open for 12 weeks in 2005. The registering physician or nurse was asked to choose one day during this time period to register all patients at the unit, with each patient representing a separate data set. All data were entered online at the palliative care unit using a web based survey generator (provided by Alstra AB, Stockholm, Sweden). 

### 2.1. Ethics

The regional ethics committee in Stockholm has given approval to PANIS for its working methods, (data gathering through cross sectional survey studies) which includes this study. Project identification code 04-716/2 and date of approval 13 October 2004.

### 2.2. Statistical Analysis

Data from the survey generator were transferred to Excel and SPSS version 17 for analysis. Patient characteristics are described using means, standard deviation, and median for continuous variables. For the purpose of this study, we categorized patients as receiving: (1) ETF only; (2) PN (defined as solutions containing amino acids, fat and glucose); and (3) intravenous glucose only. Data from patients using ETF in combination with PN were analyzed as part of the PN group. Differences in proportions were analyzed with Chi-Square tests and *t*-tests. For between group analyses, we used Kruskal-Wallis tests followed by a *post-hoc* Mann-Whitney *U*-test. Statistical significance was set at *p* ≤ 0.05 for all analyses and all tests were two-sided.

In the analyses of indications and perceived benefit of treatment, the included patients were divided into the following three groups based on expected survival: (1) palliative care with predicted survival time ≤one month; (2) palliative care with predicted survival >one month; (3) potentially curable patients.

## 3. Results

Data was received from 32 of the 42 units in the PANIS research network, representing 20 PIU and 26 PHC services, with some units including both services. Data from 1083 patients with heterogeneous cancer diagnoses were included in the analyses, with between one and 60 patients included from each service.

### 3.1. Patient Characteristics and Prevalence of Use of Enteral Nutrition, Parenteral Nutrition and Intravenous Glucose

Data from 500 men (46%) and 583 women (54%) were included in the study. No significant differences were found between PHC and PIU services regarding sex, although PHC patients were significantly younger (*p* = 0.001) than those in PIU. Gastrointestinal malignancies were the most prevalent diagnoses in both PIU and PHC services. In Table 1 demographic data are presented in relation to care setting and use of ETF, PN and glucose.

Thirteen percent (*n* = 143) of the included patients received ETF, PN or glucose. Ninety-four patients received PN, 26 intravenous glucose and 23 ETF. No statistically significant differences were found regarding the prevalence of PN and ETF between PHC services (10%) and PIU (14%). However, the use of glucose was significantly more common (*p* < 0.001) in PIU (8%) than in PHC services (1%) (Table 1).

### 3.2. The Prevalence and Use of Enteral Tube Feeding, Parenteral Nutrition and Intravenous Glucose in Palliative In-Patient Units

As shown in Table 1, 22% of the 192 PIU patients received ETF, PN or glucose, with PN being most common (12%). 

#### 3.2.1. Enteral Tube Feeding

Three of the four patients receiving ETF-only in a PIU had head-and-neck cancer (Table 1). All four patients had received ETF through a percutaneous endoscopic gastrostomy tube for 6–7 days during the week prior to registration. All four patients receiving ETF-only were reported from PIUs in the Stockholm area. 

#### 3.2.2. Parenteral Nutrition

As shown in Table 1, gastrointestinal and gynecological malignancies were the most common diagnoses among the 23 patients receiving PN in PIUs. Seventeen of these patients had a subcutaneous venous access port for PN administration and three used an infusion pump. Two-thirds (*n* = 15) received PN-infusions for 5–7 days during the week prior to registration; seven patients had received PN-infusions on 3–4 days during the past week. Vitamins and minerals were added to the PN solutions for 17 of the patients. Two of the patients received PN in combination with ETF.

**Table 1:** Characteristics of patients by palliative care setting and use of enteral tube feeding, parenteral nutrition and intravenous glucose (*n* = 1083).

Palliative In-Patient Units						Palliative Home Care Services				
	Total (%)	ETF (%)	PN (%)	Glucose (%)	No ANH (%)	Total (%)	ETF (%)	PN (%)	Glucose (%)	No ANH (%)
	*n* = 192	*n* = 4	*n* = 23	*n* = 16	*n* = 149	*n* = 891	*n* = 19	*n* = 71	*n* = 10	*n* = 791
	(100)	(2)	(12)	(8)	(78)	(100)	(2)	(8)	(1)	(89)
Sex *n* (%)										
Male	81 (42)	4 (100)	10 (44)	8 (50)	59 (40)	419 (47)	9 (47)	28 (39)	3 (30)	379 (48)
Female	111 (58)	0 (0)	13 (56)	8 (50)	90 (60)	472 (53)	10 (53)	43 (61)	7 (70)	412 (52)
Age										
Mean	71	72	65	70	71	67	62	62	63	68
(Range)	(31–93)	(59–85)	(40–84)	(41–90)	(31–93)	(16–100)	(41–82)	(22–92)	(25–87)	(16–100)
Type of cancer *n* (%)										
Breast	18 (9)	-	1 (4)	3 (19)	14 (10)	115 (12)	-	4 (6)	1 (10)	110 (14)
Lung	30 (16)	-	1 (4)	2 (12)	27 (18)	105 (12)	-	6 (8)	-	99 (13)
Prostate	13 (7)	-	-	1 (6)	12 (8)	121 (14)	-	2 (3)	1 (10)	118 (15)
Gynecological	28 (15)	-	7 (30)	3 (19)	18 (12)	80 (9)	-	17 (24)	1 (10)	62 (8)
Gastrointestinal	49 (26)	-	10 (44)	4 (25)	35 (23)	228 (26)	5 (26)	32 (45)	6 (60)	185 (23)
Hematological	10 (5)	-	1 (4)	1 (6)	8 (5)	53 (6)	-	2 (3)	-	51 (6)
Other	44 (23)	4 (100)	3 (13)	2 (12)	35 (23)	189 (21)	14 (74)	8 (11)	1 (10)	166 (21)
- Head Neck	-	3 (75)	-	-	-	24 (3)	13 (68)	2 (3)	-	9 (1)
Patients from Stockholm area	114 (59)	4 (100)	15 (65)	8 (50)	87 (58)	506 (57)	15 (79)	55 (77)	4 (40)	432 (55)
Patients from other parts of Sweden	78 (41)	0 (0)	8 (35)	8 (50)	62 (42)	385 (43)	4 (21)	16 (23)	6 (60)	359 (45)

ANH: Artificial nutrition/hydration; ETF: Enteral tube feeding; PIU: Palliative in-patient unit; PHC: Palliative home care; PN: Parenteral nutrition.

#### 3.2.3. Intravenous Glucose

Gastrointestinal and gynecological malignancies were the most common diagnoses in the 16 patients receiving glucose in a PIU (Table 1). Five patients had a subcutaneous venous access port for administration and seven patients received glucose via peripheral catheters. Seven of the 16 patients had received glucose-treatment for 6–7 days during the week prior to registration, three had received glucose for 3–4 days and six had received glucose for 1–2 days.

### 3.3. The Prevalence and Use of Enteral Tube Feeding, Parenteral Nutrition and Intravenous Glucose in Palliative Home Care Services

Eleven percent (*n* = 100) of the 891 patients enrolled in PHC services received ETF, PN or glucose with PN being the most common (8%). 

#### 3.3.1. Enteral Tube Feeding

As shown in Table 1, 13 of the 19 patients in PHC services on ETF had head-and-neck cancer. A percutaneous endoscopic gastrostomy was the most common mode for ETF-administration (*n* = 16). All patients on ETF were reported to have received daily feeding. The patient and/or family members administered the feedings in the majority of the cases (*n* = 16); three patients had assistance from the palliative home care team for ETF-administration. 

#### 3.3.2. Parenteral Nutrition

Gastrointestinal and gynecological malignancies were the most common diagnoses among the 71 patients receiving PN in PHC services. Use of PN was significantly more common (*p* < 0.001) in PHC services in the urban Stockholm region (11%) compared to other parts of the country (4%). The prevalence of PN in the two other major urban areas in Sweden was lower than average (2%). In general, PN was administered with assistance from a nurse in the PHC team; in two cases the patient and/or family administered the treatment themselves. The number of days when PN-treatments were administered during the week prior to registration varied. Over half of these patients received PN-treatment on 5–7 days (*n* = 40), 23 received treatment on 3–4 days and three patients 1–2 days/week. For the majority of patients (*n* = 62) vitamins and minerals were added to the PN-solution. 

#### 3.3.3. Intravenous Glucose

Six of the 10 patients who received glucose-only had gastrointestinal-malignancies (Table 1). In all cases, a nurse from the PHC team administrated the solution. Four patients received glucose on 5–7 days, 2 on 3–4 days and four on 1–2 days during the week prior to registration.

### 3.4. Enteral Tube Feeding, Parenteral Nutrition and Intravenous Glucose in Relation to Predicted Survival Time

#### 3.4.1. Enteral Tube Feeding

As shown in Table 2, the most common indication for ETF was difficulty chewing and/or swallowing regardless of predicted survival. Weight loss was reported as the second most common indication. Nine of the 23 patients with ETF had some degree of oral intake with five patients able to eat solid food. Six patients on ETF were also prescribed oral nutritional supplements. The registering physician or nurse reported that ETF treatment was beneficial for all but one of these 23 patients. This patient had a predicted survival time ≤one month. 

#### 3.4.2. Parenteral Nutrition

The indications for PN-treatment are shown in Table 2. Weight loss (*n* = 52) and appetite loss (*n* = 47) were the most common indications reported among the 94 patients regardless of predicted survival time. Nausea and vomiting were reported as an indication for PN for 29 patients. Semi-obstruction/obstruction was the indication for PN for 17 of the 86 patients in a palliative phase. Nutritional support during anti-tumoral treatment was reported to be an indication in 22 of the patients in a palliative phase. Four of the 20 patients with PN whose survival time was predicted to be ≤one month, had requested PN him/herself and in another case a significant other had suggested PN. Seventy-five of the 86 patients (87%) in palliative stages receiving PN were reported as also having some oral intake, with 31 of these patients only able to consume liquids. Oral nutritional supplements were reported to be used by 40 patients receiving PN in a palliative phase. The PN-treatment was deemed to be beneficial in three quarters of the patients in palliative phase, *i.e.*, in eight of the 20 patients with predicted survival ≤one month, and in 57 of the 66 patients with predicted survival time >one month (Table 2). There were no statistically significant differences in the distribution of artificial nutrition (ETF and PN) between the different survival groups.

#### 3.4.3. Intravenous Glucose

The most common indications for receiving intravenous glucose among the patients in a palliative phase were dehydration and nausea/vomiting. In the majority of these cases, staff deemed this treatment to be beneficial, even for patients with predicted survival ≤one month. 

**Table 2:** Oral intake, indication * and evaluation of patients receiving enteral tube feeding, parenteral nutrition and intravenous glucose in relation to expected survival.

	Enteral Tube Feeding Only (*n* = 23)			Parenteral Nutrition (*n* = 94)			Intravenous Glucose (*n* = 26)		
Stage of Disease	Palliative Care (*n* = 19)		Potentially Curable (*n* = 4)	Palliative Care (*n* = 86)		Potentially Curable (*n* = 8)	Palliative Care (*n* = 25)		Potentially Curable (*n* = 1)
	Predicted Survival Time			Predicted Survival Time			Predicted Survival Time		
	≤1 Month	>1 Months		≤1 Month	>1 Months		≤1 Month	>1 Month	
	(*n* = 3)	(*n* = 16)		(*n* = 20)	(*n* = 66)		(*n* = 14)	(*n* = 11)	
Oral intake	1	7	1	15	60	7	12	11	1
Food	-	5	-	9	35	5	3	5	-
Liquids only	1	2	1	6	25	2	9	6	1
Use of oral nutritional supplements	-	5	1	10	30	3	2	5	1
Indication for artificial nutrition									
Loss of appetite	-	1	-	13	32	2	4	4	-
Weight loss	-	7	2	12	39	1	2	-	-
Gastrectomy/short bowel	-	5	-	-	5	-	1	-	-
Dehydration	-	-	-	4	3	1	6	6	1
Diarrhea	-	-	-	1	3	-	-	1	-
Semi obstruction/obstruction	-	-	-	4	13	-	7		
Nausea, vomiting	-	-	-	4	23	2	7	3	1
Difficulties in chewing and/or swallowing	3	12	4	6	16	4	4	1	-
Nutritional support during anti-tumoral treatment	-	1	2	4	18	3	-	-	-
Suggestion from acute hospital	-	2	-	7	14	5	2	-	-
Suggestion from patient	-	-	-	4	16	1	1	-	-
Suggestion from significant other	-	-	-	1	6	-	1	-	-
Pre/postoperative support	-	1	-	-	1	1	-	-	-
Large losses from stoma	-	-	-	-	1	-	-	-	-
Other indications	-	-	-	-	-	-	4	2	1
Benefit of artificial nutrition according to team assessment									
Yes	2	16	4	8	57	7	12	9	1
Doubt benefit	-	-	-	10	9	1	1	1	-
No	1	-	-	2	-	-	1	1	-

* More than one indication could be chosen.

## 4. Discussion

In this national explorative cross-sectional study of 1083 cancer patients from 20 PIU and 26 PHC services in Sweden, we found that over 13% of patients received ETF, PN or intravenous glucose. PN was the most common treatment in both types of palliative care settings. Weight loss and appetite loss were the most common reported indications for PN, in line with results from previous studies [1,17,19]. PN was generally combined with oral intake and oral nutritional supplements. PN was also significantly more common in PHC services in the urban Stockholm area than in the rest of the country.

We found that while gastrointestinal and gynecological cancer diagnoses were most common among PN users, semi-obstruction/obstruction was reported as an indication for PN in only 17 of the 84 patients in a palliative phase. The European Society for Clinical Nutrition and Metabolism (ESPEN) [5] generally recommends ETF as the first choice of nutritional support for patients with insufficient oral intake and a functioning gastrointestinal tract [5]. We did not investigate if EFT had been given prior to the introduction of PN, however it is likely that many patients receiving PN had some kind of intestinal failure which was not only related to mechanical problems of intestinal transit, but to a combination of anorexia and early satiety. 

We found that the number of cancer patients with ETF was small, did not differ between PHC and PIU, and largely consisted of patients with difficulties in chewing and/or swallowing due to head-and-neck malignancies. These results correspond to our earlier findings from the Stockholm region [17], indicating that ETF in Sweden is rarely used for patients in palliative cancer phases with limited oral intake, for reasons other than chewing and/or swallowing difficulties. 

Almost 60% of the 891 participants from PHC services were recruited from the Stockholm area. The prevalence of PN and ETF use among these urban patients corresponded with that found in our previous interview study of over 600 patients enrolled in PHC teams in Stockholm [1,17]. Interestingly, the present study indicates differences in PN-use in different parts of Sweden with significantly higher use of PN in PHC teams in the Stockholm area compared to other areas of Sweden. Possible explanations might include different treatment traditions and attitudes towards the benefit of nutritional support in palliative phases, but it might also reflect differences in predicted survival of patients enrolled in different units. We had expected that greater distances to the patient’s home for PHC teams working in larger catchment areas might be another reason, as this could make administration more difficult. However, the low prevalence of PN in the two other large urban areas in Sweden does not support this explanation. Although the data was collected in 2005, clinical reports from the research network indicate an unchanged prevalence of artificial nutrition in specialized palliative care.

In line with our previous studies [17,19] almost all patients living at home with ETF managed its administration within the family, whereas patients with PN received help from a PHC team nurse. This appears to differ from routines in many other countries where the standard procedure is that patients and/or families are responsible for both ETF and PN administration. Training the patient and/or family members to administer PN may increase the patient’s autonomy and might be valuable for some patients. However, for patients in palliative cancer phases, frequent home visits from the PHC staff to administer PN have been described as a particularly positive part of the PN experience, due to the sense of security afforded to both patient and family [19]. If limited resources and long distances in rural areas are obstacles for nurses to assist with administration of PN, the development of teaching programs for self-administration might prove valuable for patients who could potentially benefit from PN. 

The lack of equivalent data makes direct comparisons between Sweden and other countries difficult. Burnette *et al.* [13] raises questions about the role that economic and cultural factors might have in influencing decision-making regarding nutritional support. In Sweden, treatment with PN involves no out-of-pocket cost to patients enrolled in PHC teams. The palliative care team does not have to consider family income or private insurance when suggesting PN. Also, registered nurses in Sweden are licensed to insert IV cannulas and administer infusions, facilitating this type of treatment by professionals, especially in the home. Different countries have different approaches to supportive and palliative care; might it be then that, given the facts above, some Swedish palliative teams are more likely to consider this treatment option?

Distress for both family and patients when a patient faces severe anorexia and weight loss has been demonstrated by several studies [18,22,23,24]. This distress can have a negative impact on family life and create tensions around mealtimes [18,23,24]. In an interview study of the experiences of advanced cancer patients prior to initiation of HPN, patients reported wanting to eat, but being unable to do. Family members experienced feelings of powerlessness as they could not help the patient to eat [18]. This inability to eat has been described as “hitting the wall” [25].

Eating difficulties and weight loss were the most commonly reported indications for PN in this study. Our clinical experience is that the close connection between food and life is often referred to by staff as a reason for patients’ and families’ requesting PN in a late palliative phase. This can be experienced as an ethical challenge for both staff, patients and family as PN is expensive, demanding, time consuming and potentially risky. In the present study, the indication for PN in 20 of the 86 PN users in a palliative phase was reported as being the patient’s suggestion, and in 21 cases PN was based on suggestions by hospital staff. Family was reported as suggesting PN for only seven of the 86 patients. This contradicts a common assumption often found among clinicians, *i.e.*, that requests from relatives are one of the most common indications for starting PN. 

Initiating ETF and PN is often presented as an ethical challenge for clinicians [26,27]—will nutritional support be beneficial or futile? One of the challenges is to accurately estimate expected survival time. In this study, we divided patients into three groups based on expected survival. Although there are few patients in the group of potentially curable patients who have a long-lasting response, as they are deemed potentially curable by the oncologists, and they receive aggressive treatment. In Sweden, these patients are referred to palliative care services (mainly home care) for supportive treatment when adverse effects of antitumoral treatment are encountered. As there is no separate organization caring for this patient group. Patients with ovarian cancer or colorectal cancer are examples of patients in this group. In accordance with the working method of the PANIS network and in line with the WHO definition of palliative care, these patients, when being enrolled at the palliative care unit, were registered in the study. 

Other challenges include achieving agreement between professionals, patients and family members on treatment goals and also being able to correctly evaluate nutritional support, especially when nutritional status and physical function are not expected to improve. PN is generally recommended only for patients with expected survival longer than 2–3 months [5]. For patients with shorter life expectancy and refractory cachexia, the burdens and risks of artificial nutrition are likely to outweigh potential benefits [4]. Our finding that 20 patients with an expected survival of ≤1 month received PN suggests that clinical decisions were not based on available evidence [5] alone, but that ethical and psychological considerations also contributed to decision-making. To better understand this, it would be interesting to further investigate why PN is used in end-of-life. A recent review of the use of artificial nutrition and hydration in the last week of life found only one study assessing the effect of the treatment on symptoms and quality of life, with no reported benefit in comfort [15]. In the present study, staff was asked to assess the benefit of the nutritional support. However, no criteria were provided for this assessment, thereby limiting further understanding and indicating that the results should be interpreted with caution. It might be expected that use of glucose would be more common than use of PN in patients with short predicted survival. On the contrary, the results from our study showed that the use of PN was more common than glucose. 

Clinically useful methods to evaluate the effects and eventual benefits of PN treatment in the palliative phase are needed to help physicians, nurses and dietitians in decision-making. Regular assessment of functional level is recognized as providing more relevant, robust and patient-centered information about effects of nutritional interventions than muscle mass *per se* [28]. The same applies for objective analyses of specific blood parameters, measurement of weight, and BMI. As the goal of palliative care is to reduce symptoms and improve quality of life for both patients and family, evaluation of subjective goals and experiences, e.g., effects on family and social life, appetite, sleep, as well as distress from nutritional interventions and nutritional intake, is most important [19]. Recently Baxter *et al.* [29] developed a questionnaire to assess quality of life in adult patients with chronic intestinal failure and long-term PN. A similar questionnaire could be valuable for patients with malignant diseases receiving short-term-PN, as this would facilitate standardized evaluation of nutritional support, which includes the patient perspective. 

As mentioned in the introduction, recently published consensus definitions of cancer cachexia state that cachexia cannot be fully reversed by conventional nutritional support [4]. However, patients today live longer with incurable diseases and the palliative phase may include several cycles of anti-tumoral treatment over many years. Earlier data from PANIS based on 16 Swedish palliative care units [12] reported that one third of the >500 patients studied received palliative chemotherapy. As the palliative phase has become more extended, it has been suggested that better integration of supportive and palliative care, including proper nutritional support, should be initiated in earlier palliative phases when the metabolic changes associated with cachexia are less prominent [30,31]. In our study, nutritional support during anti-tumoral treatment was reported as an indication for PN in a quarter of the PN users in a palliative phase. This indicates a need to systematically evaluate the effects and eventual benefits of supportive ETF and PN in combination with anti-tumoral treatment in palliative phases.

This cross-sectional study, using well-established working procedures from the PANIS network, included patients from a large number of PIU and PCH services across Sweden with a high participation rate resulting in a large sample size. All participating units were explicitly requested to register all patients on the unit whether or not they received nutritional treatment or glucose. The design of the web-based survey ensures full completion of each set of patient data, with no missing data for individual patients. According to the working methods of PANIS, more in-depth demographical data and information about the research question is collected only for patients who are actually receiving the specified treatment. This means that information about palliative phase/potential curability was only registered for patients receiving nutritional support or glucose. It was therefore not possible to calculate the proportion of patients in different phases for the entire material of 1083 registered patients. Accordingly, data regarding the patient’s nutritional status, anti-tumoral treatment and predicted survival time was only registered in patients receiving ETF, PN or intravenous glucose, and limits comparisons between groups. Despite the limits of this cross-sectional approach, this study nevertheless contributes valuable new insights in an under-researched area.

This study again emphasizes the need for continued research in this area. Documentation of cachexia using the recently developed definition of its stages [4] is one important aspect for future study. Another important area is the development of comprehensive cachexia assessment tools to identify the cachexia stages in clinical practice, as such tools are still lacking [32]. Also, classification of whether weight loss and nutritional problems are due to cachexia or secondary causes is important. This will help facilitate the differentiation of outcomes of nutritional support for patients in different palliative phases and will assist in finding the most appropriate treatment for patients in different phases.

## 5. Conclusions

In conclusion, this national study provides unique data about the use of ETF, PN and intravenous glucose among patients enrolled in specialized palliative care in Sweden, which can serve to guide future research. Treatment with PN was found to be more common than ETF, but we found significant differences in the use of PN within the country. These differences, both between urban and rural regions, as well as in use of PN among different urban palliative care settings, demand further investigation. PN was found to be mainly initiated when the patient had problems with weight and appetite loss but still had oral intake, indicating a use of PN extending beyond that, which is traditional in patients with obstruction/semi obstruction. This approach might be particularly valid when PN is not exclusive but is given as a supplement a few times a week. It is important to further investigate the potential values and disadvantages of artificial nutrition for patients enrolled in palliative care. 

## 6. Implications

As there is limited data about the prevalence and indications for artificial nutrition, this study adds important information for health care professionals. The results of our study can be used to discuss and further refine the use of artificial nutrition in specialized palliative care, to help clinicians in decision-making.
